# Assessment of the imaging quality and heterogeneous predictive value of readout-segmented echo planar imaging for diffusion-weighted rectal MRI

**DOI:** 10.3389/fonc.2025.1457238

**Published:** 2025-07-30

**Authors:** Mingrui Song, Yichuan Liang, Huiying Wang, Lei Shi, Quanliang Mao, Haonan Zhao, Zhehao Zhang, Aijing Li, Yuning Pan

**Affiliations:** ^1^ Department of Radiology, The First Affiliated Hospital of Ningbo University, Ningbo, Zhejiang, China; ^2^ Health Management Center, Shandong Chronic Disease Hospital, Qingdao, Shandong, China; ^3^ Department of Radiology, Ningbo No. 2 Hospital, Ningbo, Zhejiang, China

**Keywords:** imaging quality, readout-segmented echo planar imaging, single-shot echo planar imaging, diffusion-weighted imaging, Ki-67, hypoxia inducible facor-1α, predictive value

## Abstract

**Objectives:**

To qualitatively and quantitatively compare the image quality of readout-segmented echo planar imaging (rs-EPI) and single-shot echo planar imaging (ss-EPI) for diffusion-weighted (DWI) rectal MRI, as well as the heterogeneous predictive value of the apparent diffusion coefficient (ADC) values obtained by the two DWI techniques.

**Methods:**

The rs-EPI and ss-EPI images were subjectively assessed for lesion sharpness, display of normal structure, overall image quality, geometric distortion, and anatomical differences. The signal-to-noise ratio (SNR), contrast ratio (CR), contrast-to-noise ratio (CNR), and ADC values were objectively compared. Pearson’s correlations and ROC analysis were used to explore the relationships of ADC values obtained by the two techniques and nucleus related antigen (Ki-67) and hypoxia inducible factor-1α (HIF-1α).

**Results:**

Eighty patients with rectal cancer (RC) were included. Lesion sharpness, normal structure display, overall image quality, geometric distortion and anatomical structure differences in the rs-EPI DWI group were higher than in the ss-EPI DWI group (*P*<0.001). SNR, CNR and CR in the rs-EPI DWI group were higher than in the ss-EPI DWI group (*P*<0.001). ADC values were not different. ROC analysis showed that the area under the curve (AUC) of high Ki-67 and HIF-1α expression levels as predicted by the average ADC of ss-EPI and rs-EPI DWI were 0.82 (95%CI: 0.72-0.92), 0.77 (95%CI: 0.67-0.88), and 0.81 (95%CI: 0.72-0.91), 0.82 (95%CI: 0.72-0.91), respectively, with similar predictive values between the 2 techniques (*P*=0.23, 0.75).

**Conclusion:**

rs-EPI DWI can improve image quality and the ADC value is associated with pathologic markers of tumor aggression.

## Introduction

Colorectal cancer is ranked as the third most common cancer worldwide, with rectal cancer (RC) accounting for 30-35% of colorectal cancer cases (1). A comprehensive understanding of the heterogeneity of RC prior to treatment can help establish a better management approach (2). Numerous factors have been reported as indicators of tumor aggression and prognosis pathologic markers for RC, including the Ki-67 index, which is associated with tumor proliferative activity (3), and hypoxia inducible factor-1α (HIF-1α) expression, which is associated with the tumor hypoxic microenvironment (4). However, measuring these markers requires invasive pathological biopsies (5). Moreover, the specimens obtained by a single biopsy may not represent the entire internal environment of the tumor (6). Therefore, it would be of great clinical value to have a non-invasive imaging method that can accurately reflect the entire tumor proliferation status and tumor hypoxic microenvironment.

Magnetic resonance imaging (MRI) is a noninvasive standard method for the diagnosis and evaluation of RC due to its excellent tissue resolution (7). The apparent diffusion coefficient (ADC) is obtained using diffusion-weighted imaging (DWI) and can quantitatively evaluate the spread intensity of the tumor and indirectly reflect the cell structure and microscopic changes of the tissue (8). Abnormal proliferation and hypoxia of tumors can lead to changes in cell structure, so we speculate that ADC values may be related to tumor proliferation and hypoxia (9). In clinical practice, single-shot echo planar imaging (ss-EPI), which has the advantage of fast acquisition speed. is often used in DWI. However, using a single excitation to fill the entire K-space may produce artifacts at the intersection of tissues due to magnetic sensitivity changes and the highest spatial resolution that can be achieved is relatively low. In a 3.0 T magnetic field environment, magnetic sensitivity artifacts are more serious, and the acceleration of T2* attenuation may result in blurred images. In areas with complex structures such as the pelvic floor, imaging artifacts and severe deformation are likely to occur due to the different magnetic susceptibilities of the various soft tissue - rectal air and soft tissue - rectal air and soft tissue - pelvic interface, resulting in inaccurate ADC measurements (10). The RESOLVE DWI using readout-segmented echo planar imaging (rs-EPI) technique can not only improve image quality and reduce magnetic sensitive artifacts by reducing TE and echo interval time, but can also reduce specific absorption ratio (SAR) value and phase artifacts generated by motion (11). Several studies have shown that the use of rs-EPI can improve image quality and diagnostic performance in brain, breast, kidney, and liver imaging (12–15). However, the performance of this technique in RC diagnosis has not been studied. In addition, the relationship between the ADC values obtained by rs-EPI and Ki-67, HIF-1α values have not yet been explored (16, 17). Therefore, the primary aim of this study is to explore the impact of the rs-EPI technique on imaging quality and ADC measurement. The secondary aim of this study is to determine the relationship between ADC values obtained by rs-EPI and Ki-67 or HIF-1 α values.

## Materials and methods

### Study subjects

**Figure 1:**
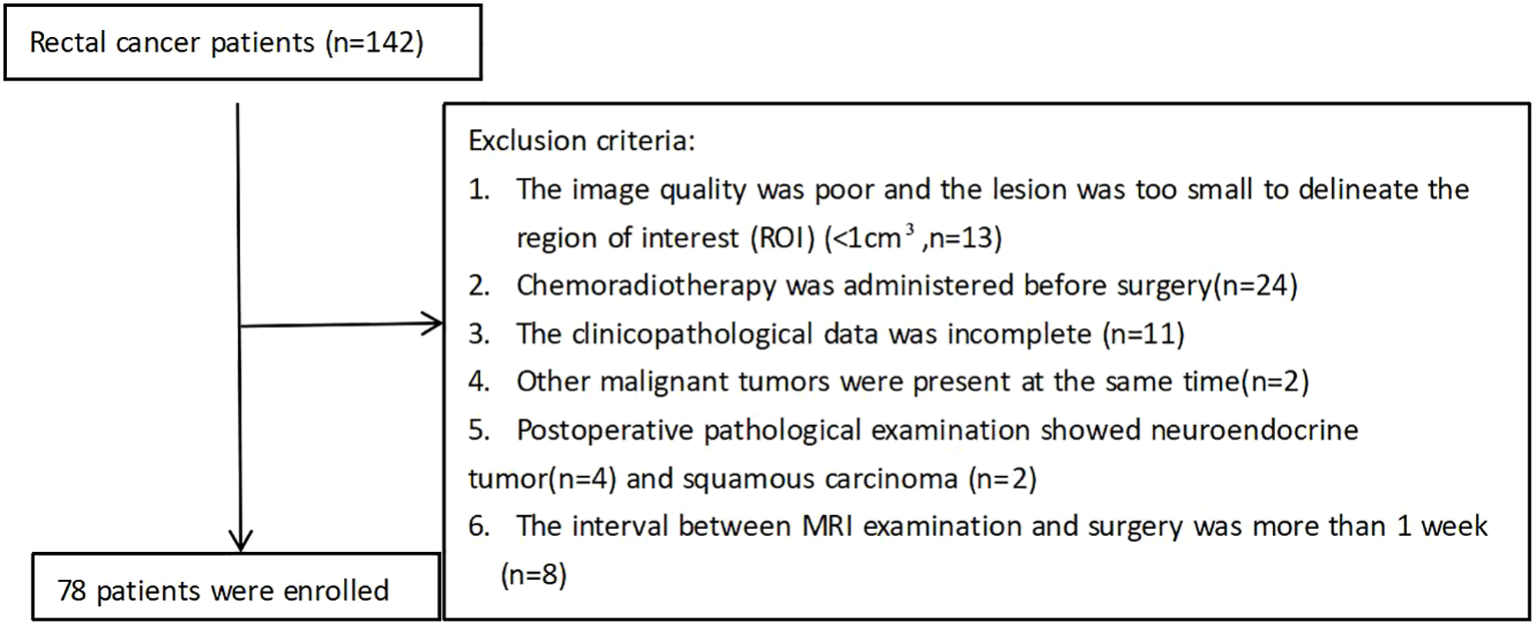
Patient inclusion flowchart.

The study was approved by the institutional review board of our hospital and informed consent was obtained from all patients prior to study enrollment (approval number: 2022- R01025). Clinicopathologic data of patients with RC who were admitted to the hospital between March 2023 and December 2023 were retrospectively analyzed. All patients underwent a colonoscopy before the operation. The final surgical pathological results were confirmed by a pathologist with more than five years of experience in digestive tract tumors. All patients underwent 3.0T MRI and DWI sequences were performed using two techniques: rs-EPI and ss-EPI. The specific exclusion criteria are shown in **Figure 1**.

### MRI examination protocols

All patients were examined in a supine head position using a Siemens 3.0T MR scanner (MagnetomVida, SIEMENS Healthcare, Germany) and an 18-channel pelvic phased-alignment ring, scanning the iliac bone to the anal margin. All patients were routinely fasted for more than 4 hours before MRI examination. Gadopentetate dimeglumine was injected intravenously with an Urich (Germany) double-channel high-pressure syringe (Beijing Beilu Pharmaceutical Co., LTD., dose: 0.1mmol/kg, rate: 2.0 mL/s), followed by a 20mL saline injection at the same rate. The plain scan sequences included T1-fl(fast low angle shot)3d, the cross-sectional fat suppressed T2WI, the DWI was performed by rs-EPI and ss-EPI, and the B-value included two sequences: 50 and 1000 s/mm^2^. The DWI parameters are shown in **Table 1**. The ADC value is automatically generated by the post-processing sub-station.

**Table 1:** Imaging parameters of rs-EPI and ss-EPI DWI.

Sequence parameters	RESOLVE (rs-EPI)	ss-EPI
TR/TE (ms)	5600/54	5600/54
Phase encoded direction	Forward and backward	Forward and backward
FOV (mm)	380×192	380×192
Matrix	190×96	190×96
Layer thickness (mm)	4	4
slice gap (%)	20	20
Acquisition time (min:s)	3 min 13 s	2 min 42 s
Reversal time (ms)	210	210
Fat suppression	SPIRE	SPIRE
b value (s/mm^2^)	0,1000	0,1000
Segments number	5	1

DWI, diffusion weighted imaging; RESOLVE, readout-segmented acquisition; ss-EPI, single-shot echo planar imaging; TR, repetition time; TE, echo time; FOV, field of vision.

### Imaging analysis

Subjective imaging quality score: Two radiologists with more than 5 years of experience in gastrointestinal MRI diagnosis reviewed and evaluated two sets of transversal DWI images (rs-EPI and ss-EPI). They were blinded to the histopathological diagnosis and DWI Sequence type. The images were evaluated for lesion sharpness, normal structure display, overall image quality, geometric distortion, and anatomical structure difference. A 5-point scoring system was used to score the images (12). A score of 5 points represented the best image quality while a score of 1 represented very poor image quality.

Objective image quality score: The ADC measurements were performed on images produced using Syngo workstation (Siemens, Germany). Measurements of ADC values were performed by two senior radiologists (YN Pan and AJ Li) with more than 10 years of experience in this field.

The radiologists selected three regions of interest (ROIs) in the plane of maximum tumor size on the DWI image. The same ROI was then automatically overlaid on the ADC images. Each ROI had an area ≥4 mm^2^. The average value of the three ROI areas was taken as the final result.

**Figure 2:**
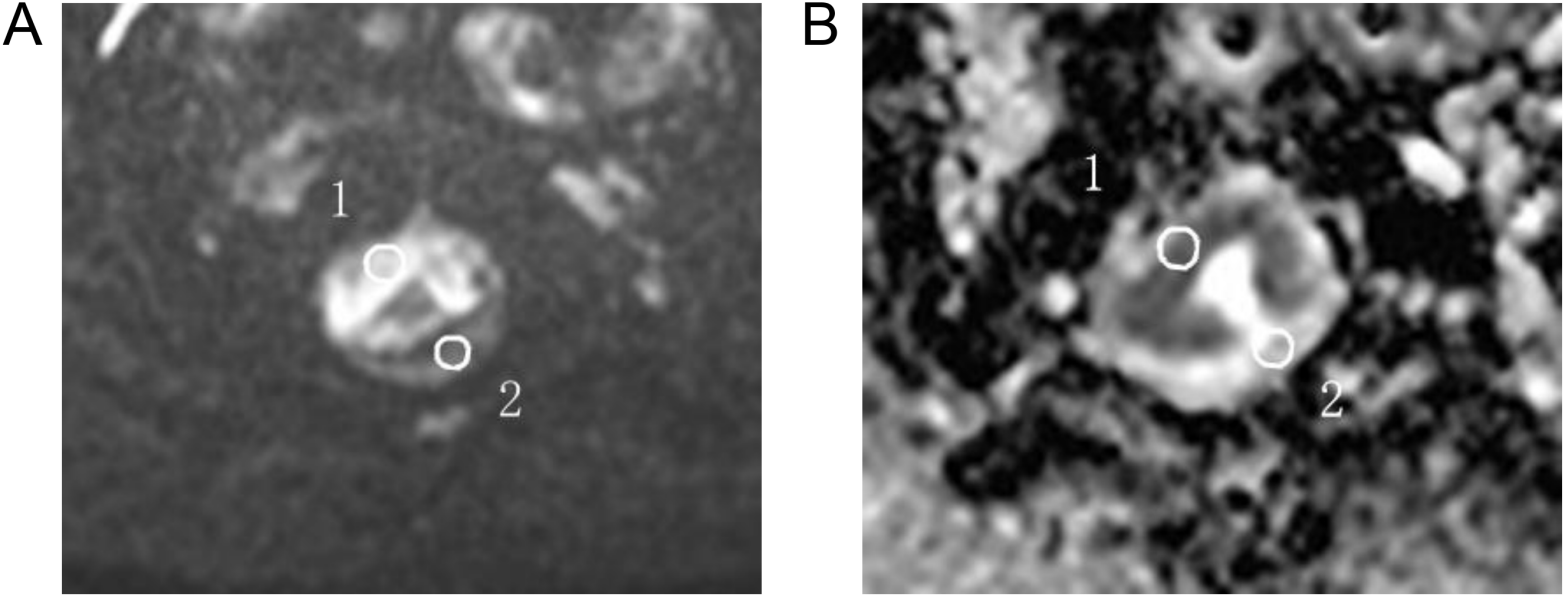
For ADC measurements, the ROI was manually plotted **(A)** and then copied onto the ADC map **(B)** 1 for tumor measurements and 2 for normal intestinal wall measurements.


The ROI was replicated onto the ADC maps of the rs-EPI and ss-EPI DWI imaging (**Figure 2**). The ROI of normal tissue was plotted as far away from the tumor as possible and the ROI of the lesion included the entire tumor area as much as possible, but avoided areas that displayed necrosis, cystic degeneration, and bleeding. Objective evaluation parameters included: I. signal intensity (SI) of the normal rectal wall; II. noise (standard deviation, SD), representing the background air; III. signal-to-noise ratio (SNR), defined as the ratio of the mean signal strength of the lesion (SI lesion) to the mean signal strength of normal tissue on the DW image (SI normal); IV. contrast-to-noise ratio (CNR), defined as the ratio of the absolute value of the signal intensity of the lesion and normal tissue to the noise (SD); VI. contrast ratio (CR), defined as the ratio of the signal strength of the lesion to that of the normal rectal tissue. VII. diameter of the lesion on the DWI (using the enhanced image as the reference standard), and the average signal strength and standard deviation for each ROI measured. The SNR, CR and CNR were calculated using the following formula (18):

$$\text{SNR}=\text{S}{\text{I}}_{\text{Normal}\;\text{tissue}}/\text{S}{\text{D}}_{\text{Background}};$$

$$\text{CR}=\text{S}{\text{I}}_{\text{Lesion}}/\text{S}{\text{I}}_{\text{Rectum}};$$

$$\text{CNR}=|\text{S}{\text{I}}_{\text{Lesion}}-\text{S}{\text{I}}_{\text{Rectum}}|/\text{S}{\text{D}}_{\text{Background}}$$

### Immunohistochemistry for Ki-67 and HIF-1α

The immunohistochemistry tissue is derived from surgical specimens. Ki-67 expression was considered positive when the nuclei were stained brown. The percentage of Ki-67 positive cells per 1000 cells observed at ×40 was recorded, and semi-quantitatively categorized as low (≤10% immunopositive cells) or high (>10% immunopositive cells). Positive expression of HIF-1α was evaluated using the appearance of a brown-yellow color upon staining with 3,3’-diaminobenzidine (DAB) and was semi-quantitatively categorized as low (≤10% of immunopositive cells) or high (>10% of immunopositive cells). All immunohistochemical operations and interpretations were carried out by full-time pathologists with more than 5 years of experience in rectal cancer-related fields.

### Statistical analysis

Statistical analysis was performed using SPSS 25.0 software (SPSS, version 25.0, USA). The Shapiro-Wilk test was used to analyze the data normality. Data that did not conform to the normal distribution were represented by median (range interquartile, IQR) and compared with Wilcoxon signed rank sum test. Data conforming to the normal distribution were expressed as the mean ± SD and compared with the paired sample t-test. The intraclass correlation coefficient (ICC) test was used to assess agreement between the two radiologists who participated in the study evaluation, with ICC > 0.75, it indicates a relatively good degree of consistency. Wilcoxon rank sum test was used to compare the difference in image quality scores. Chi-squared tests were used to assess the association between Ki-67 or HIF-1α expression and patient characteristics. Pearson’s correlation test was used to evaluate the correlation between ADC values and HIF-1α and Ki-67 expression. Receiver Operating Characteristic (ROC) curves were used to evaluate the predictive value of the ss-EPI and rs-EPI DWI average ADC on the expression level of Ki-67 and HIF-1α. The optimal threshold was determined using the Jorden index, and the difference in diagnostic performance between ROC curves was analyzed by the Delong test. *P* < 0.05 was considered statistically significant.

## Results

### Baseline characteristics

After inclusion and exclusion criterion were applied, 78 patients were enrolled in our study. The baseline patient characteristics are shown in **Table 2**. The mean age was 55.83 ± 11.21 years and 46 (58.9%) of patients were male.

**Table 2:** Baseline characteristics of the study cohort.

Parameters	Values
Age, mean ± SD	55.83 ± 11.21
Sex	
Male	46
Female	32
Pathological type	
ductal adenocarcinoma	57
papillocarcinoma	12
mucinous adenocarcinoma	9
Degree of differentiation	
highly differentiated	17
moderately differentiated	52
poorly differentiated	9
Clinical stage	
I	31
II	14
III	33

### Subjective score of image quality

The two radiologists had optimal observer agreement in terms of lesion clarity, display of normal structure, overall image quality, geometric distortion and magnetic sensitive artifact distinction, with ICC values of 0.79-0.86 for ss-EPI and 0.85-0.89 for rs-EPI. Therefore, the result was calculated as the average score of the two physicians. Subjective score results showed that lesion resolution (3.23 ± 0.92 *vs*. 4.55 ± 0.92, *P*<0.001), display of normal structure (4.24 ± 0.87 *vs*. 4.64 ± 0.57, *P*<0.001), overall image quality (3.87 ± 0.86 *vs*. 4.42 ± 0.58, *P*=0.02), geometric distortion (3.47 ± 0.68 *vs*. 4.27 ± 0.52, *P*<0.001) and magnetic sensitivity artifacts (3.52 ± 0.56 *vs*. 4.55 ± 0.53, *P*<0.001) were statistically significant, and the scores in the rs-EPI DWI group were all significantly higher than that in the ss-EPI DWI group (**Table 3**).

**Table 3:** Subjective scores for ss-EPI and rs-EPI DWI imaging.

Parameters	ss-EPI	ICC	rs-EPI	ICC	*Z* value	*P* value
	Rad 1/Rad 2		Rad 1/Rad 2			
Lesion resolution	3.25 ± 0.91/3.20 ± 0.94	0.86	4.58 ± 0.62/4.54 ± 0.64	0.85	9.86	<0.001
Display of normal structure	4.23 ± 0.86/4.26 ± 0.88	0.83	4.66 ± 0.56/4.63 ± 0.59	0.89	13.63	<0.001
Overall image quality	3.86 ± 0.76/3.90 ± 0.92	0.79	4.39 ± 0.68/4.43 ± 0.52	0.86	7.98	0.02
Geometric distortion	3.42 ± 0.65/3.52 ± 0.75	0.85	4.25 ± 0.58/4.33 ± 0.48	0.85	13.6	<0.001
Magnetic sensitivity artifacts	3.56 ± 0.55/3.49 ± 0.58	0.82	4.59 ± 0.59/4.48 ± 0.45	0.88	23.3	<0.001

ss-EPI, single-shot echo planar imaging; rs-EPI, readout-segmented echo planar imaging; DWI, diffusion weighted imaging.

### Objective score of image quality and comparison of ADC values

The SNR, CNR and CR of the rs-EPI DWI were higher than those of the ss-EPI DWI (33.85 ± 9.02 *vs*. 97.63 ± 26.3, 1.98 ± 0.56 *vs*. 2.69 ± 0.78, 4.5 ± 0.78 *vs*. 5.69 ± 0.96, *P*<0.001). There were no significant differences in the ADC values of lesions, normal intestinal wall and maximum diameter of lesions between the ss-EPI and rs-EPI DWI sequence groups ([0.92(0.75,1.28)] *vs*. [0.90(0.73,1.26)], [1.69(0.98,2.16)] *vs*. [1.61(0.97,2.23)], and 2.88 ± 0.56 *vs*. 2.82 ± 0.59, *P*=0.76, 0.29, and 0.36) (**Table 4**).

**Table 4:** Objective scores of ss-EPI and rs-EPI DWI imaging.

Groups	SNR	CNR	CR	ADC value for lesion (× 10^−3^ mm^2^/s)	ADC value for normal intestinal wall (× 10^−3^ mm^2^/s)	Maximum diameter of lesions (cm)
ss-EPI	33.85 ± 9.02	1.98 ± 0.56	4.5 ± 0.78	0.92(0.75,1.28)	1.69(0.98,2.16)	2.88 ± 0.56
rs-EPI	97.63 ± 26.3	2.69 ± 0.78	5.69 ± 0.96	0.90(0.73,1.26)	1.61(0.97,2.23)	2.82 ± 0.59
t/Z	9.78^a^	18.50^a^	17.63^a^	0.61^b^	0.89^b^	5.69^a^
P value	<0.001	<0.001	<0.001	0.76	0.29	0.36

ss-EPI, single-shot echo planar imaging; rs-EPI, readout-segmented echo planar imaging; SNR, Signal-to-noise ratio; CNR, ontrast-to-noise ratio; CR, ontrast ratio; ADC, apparent diffusion coefficient; a: *t* value; b: *Z* value.

### The correlations of ADC values obtained by ss-EPI and rs-EPI, and Ki-67 and HIF-1α expression

The mean ADC values of the ss-EPI DWI and rs-EPI DWI groups were 0.93(0.75,1.07) ×10^−3^ mm^2^/s, and 0.91(0.73,1.01) ×10^−3^ mm^2^/s, respectively, with no statistical significance (Z=-0.18, *P*=0.86).

In the ss-EPI DWI group, the mean ADC values of the lesions with low and high Ki-67 expression levels were 1.07(0.88,1.28) ×10^−3^ mm^2^/s and 0.83(0.71,0.98) ×10^−3^ mm^2^/s, respectively, and the difference was statistical significance (Z=-4.721, *P*<0.001). In the rs-EPI DWI group, the mean ADC values of the lesions with low and high Ki-67 expression levels were 1.09(0.88,1.3) mm^2^/s and 0.83(0.70,0.98)×10^−3^ mm^2^/s, respectively, and the difference was statistical significance (Z=-4.721, *P*<0.001).

**Figure 3:**
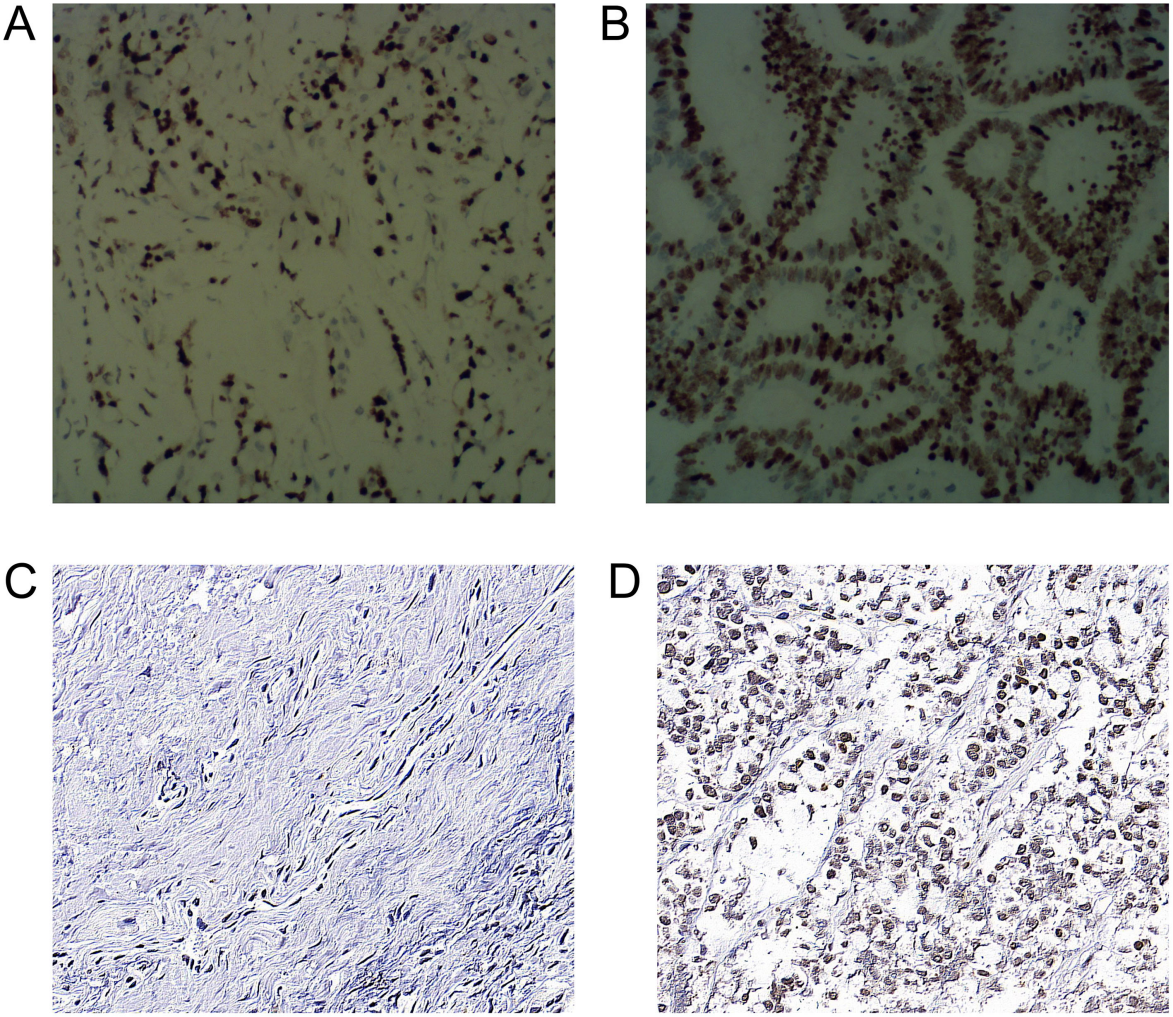
Immunohistochemical staining of Ki-67 and HIF-1α expression in rectal cancer cells **(A)** Low expression of Ki-67 in the nucleus (400×). **(B)** High expression of Ki-67 in nucleus (400×) **(C)** Low expression of HIF-1α in cytoplasm (400×) **(D)** HIF-1α in cytoplasm (400×).

In the ss-EPI DWI group, the mean ADC values of the lesions with low and high HIF-1α expression levels were 1.05(0.86,1.23) × 10^−3^ mm^2^/s and 0.83(0.71,0.92) × 10^−3^ mm^2^/s, respectively, and the difference was statistically significant (Z=-4.78, *P*<0.001). In the ss-EPI DWI group, the mean ADC values of the lesions with low and high HIF-1α expression levels were 1.07(0.81,1.26) × 10^−3^ mm^2^/s and 0.82(0.69,0.89) × 10^−3^ mm^2^/s, respectively, and the difference was statistically significant (Z=-4.67, *P*<0.001) (**Figure 3**).

ROC analysis showed that the area under the curve (AUC) of high Ki-67 and HIF-1α expression levels, as predicted by the average ADC of ss-EPI and rs-EPI DWI were 0.82 (95%CI: 0.72-0.92), 0.77 (95%CI: 0.67-0.88), and 0.81 (95%CI: 0.72-0.91), 0.82 (95%CI: 0.72-0.91), respectively, with similar predictive values between the 2 techniques (*P*=0.23, 0.75).

**Figure 4:**
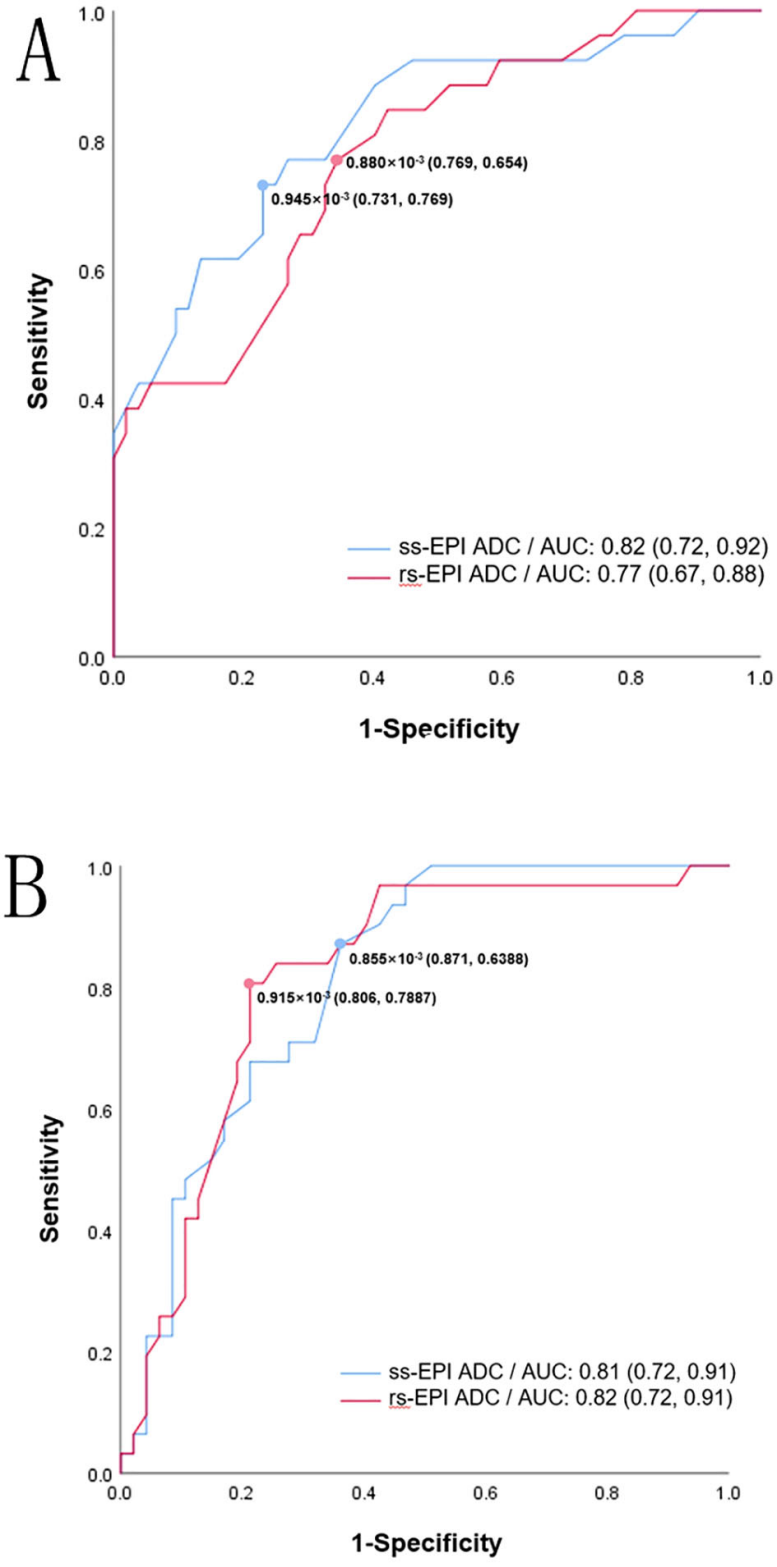
ROC curve for ss-EPI DWI and rs-EPI DWI ADC values in predicting high Ki-67 **(A)** and high HIF-1α **(B)**. The values in brackets represent the sensitivity and specificity of the cutoff values.

Both the ADC values obtained by ss-EP and rs-EPI DWI imaging were negatively associated with Ki-67 expression levels (r =−0.53, −0.46, *P*<0.001). Similarly, both the ADC values were negatively associated with HIF-1α expression levels (r =-0.53, -0.52, *P*<0.001). According to the ROC, the optimal cutoff ADC values of the ss-EPI and rs-EPI DWI to predict high Ki-67 and HIF-1α levels were 0.88× 10^−3^ mm^2^/s [Area under the curve (AUC:0.82)], 0.95 × 10^−3^ mm^2^/s (AUC: 0.77) and 0.86 × 10^−3^ mm^2^/s (AUC:0.81), 0.92 × 10^−3^ mm^2^/s (AUC: 0.82), respectively. (**Figure 4**). DeLong’s test showed that there was no statistical significance between both techniques to predict high Ki-67 expression (*P*=0.23) and high HIF-1α expression levels (*P*=0.75).

## Discussion

In clinical practice, DWI imaging often uses the ss-EPI technique, however, this technique is often susceptible to artifacts such as image blur and spatial distortion, which may also affect the accuracy of the ADC value measurement (19). Previous studies indicated that rs-EPI can not only improve image resolution and reduce magnetic sensitive artifacts by reducing TE and echo interval time, but can also reduce SAR value and phase artifacts generated by motion (20). Therefore, rs-EPI has been widely used in clinical practice. Thus far, the application of rs-EPI in the diagnosis of RC has been rarely reported on. The results of this study showed that rs-EPI DWI is superior to ss-EPI DWI in both subjective and objective image quality scores, but that the technique did not influence the measurement of ADC values, similar to what previous studies have found (21). A key feature of rs-EPI is its ability to improve image quality with reduced scan times by using fewer shots compared to traditional multi-shot techniques, which typically require longer acquisition times. However, one of the main drawbacks of this technology is the long scanning time, which is due to the need for more excitation and data acquisition cycles to fill the k-space and achieve higher-resolution imaging. Future studies will explore optimizations and provide further insight into how rs-EPI compares to multi-shot EPI, particularly in the phase encoding, where distortions are often the most pronounced (13).

ADC values represent the physical properties of the tissue, and these values can be affected by various factors, including magnetic field strength, pulse train, and b value. A stable ADC value measurement is essential for the accurate assessment of the pathological parameters of RC. In this study, the possible correlation between ADC values and pathological indicators were analyzed. We found that Ki-67 expression levels were negatively correlated with ADC values in both DWI sequences. The current findings are consistent with previous studies of other solid tumor cancers (20). The possible mechanisms are ADC values affected by a combination of intracellular and extracellular volume fractions (22). In tumor cells, the extracellular volume fraction gradually decreases with the increase in cell size, and the intracellular volume fraction gradually increases, both of which contribute to decreases in the extracellular/intracellular volume ratio (23). Therefore, the ADC value decreases with the increase in cell proliferative activity (20). Based on our findings, noninvasive ADC values can be incorporated into routine patient evaluation approaches to RC.

This study also investigated the relationship between HIF-1α expression and ADC value. Hypoxia is associated with radioinsensitivity and treatment resistance. Reliable hypoxia imaging will provide important metabolic information and hierarchical management basis for anticancer therapy. We found that the expression levels of HIF-1α was moderately negatively correlated with the ADC value in two different diffusion scan sequences. The correlation between ADC values and HIF-1α expression showed different results in different tumor types. Huang et al. concluded that ADC values showed a strong positive correlation with HIF-1α expression in hepatocellular carcinoma (24). In RC, a previous study showed that ADC was slightly negatively correlated with HIF-1α expression (25). We believe that the differences between these findings may come from different mechanisms regulating HIF-1α expression. Previous studies have shown that HIF-1α expression in soft tissue sarcomas are regulated in a non-oxygen-dependent pattern (26). In some tumors, the expression of HIF-1α in tumors is often not entirely dependent on the hypoxia level of the tumor, but is more likely to be related to the malignancy of the tumor. In contrast, HIF-1α expression in specific types of tumors is entirely dependent on oxygen-deficiency within the tumor, such as cervical and ovarian cancer (27, 28). In RC, the hypoxic environment induces glucose uptake and angiogenesis by tumor cells, leading to tumor cell proliferation and resulting in limited diffusion of water molecules. These mechanisms may explain the negative correlation of HIF-1α with ADC.

The AUC of ADC values for predicting high Ki-67 expression with both techniques were 0.82 and 0.78, respectively. To predict high HIF-1α expression, the AUCs were 0.81 and 0.82. These results suggest that the ADC values obtained by ss-EPI and rs-EPI DWI can predict the expression levels of Ki-67 and HIF-1α accurately. ADC values can be used as a non-invasive measurement method to detect cell proliferation and hypoxia, which is of great significance for anticancer treatments and stratified management of RC.

Some limitations exist in this study. First, we measured the average ADC values in tumor and normal intestinal wall areas using hand-drawn ROI regions, which may increase the possibility of sampling error. Second, we only included patients with untreated RC, so the value of DWI on treatment response remains to be studied. Third, we did not accurately correlate ADC values with excised tumor samples. That means the pathologists did not select similar ROI regions with radiologists. Fourth, the study sample was small and came from a single institution, which could lead to patient selection bias. Large multicenter studies are needed to validate the results of this study. Lastly, while high inter-reader agreement (ICC 0.79–0.89) was achieved between the two radiologists in this study, their assessments reflect subjective judgments shaped by specific training and experience. Individual scoring cannot be extrapolated to other clinicians with differing expertise or institutional protocols. Clinical application thus requires strict adherence to the study’s scoring criteria, reader training, and imaging environment; deviations may compromise reliability.

In summary, the rs-EPI DWI can improve image quality, without affecting the measurement of ADC values. In addition, ADC values were moderately negatively correlated with the expression of Ki-67 and HIF-1α, and the ADC values obtained by rs-EPI DWI and rs-EPI DWI could be used as non-invasive techniques and indicators to predict the proliferation and hypoxia of RC cells.

## Data Availability

The raw data supporting the conclusions of this article will be made available by the authors, without undue reservation.
